# Microparticles of anthropogenic origin (microplastics and microfibers) in sandy sediments: A case study from calabria, italy

**DOI:** 10.1007/s10661-024-13159-z

**Published:** 2024-10-01

**Authors:** Valentina Balestra, Federica Trunfio, Sinem Hazal Akyıldız, Paola Marini, Rossana Bellopede

**Affiliations:** 1 Department of Environment, Land and Infrastructure Engineering (DIATI), Politecnico Di Torino, Corso Duca Degli Abruzzi 24, 10129 Turin, Italy; 2 Politecnico Di Torino, Corso Duca Degli Abruzzi 24, 10129 Turin, Italy

**Keywords:** Microplastics, Microfibers, Pollution, Beaches, Micropollutants

## Abstract

Microparticles of anthropogenic origin, such as microplastics and microfibers, are pervasive pollutants in the marine environment of the world. These microparticles pollute water and can be ingested by biota; however, while microplastics are often monitored, very few studies focus on microfibers. Coastal areas, such as beaches, are more vulnerable to pollution due to their location between terrestrial and marine environments and their recreational and touristic functions. In this study, microparticle occurrence frequency was investigated along the Calabria coast, Italy, in one touristic beach in comparison with an unpopular one. High amounts of microparticles of anthropogenic origin were found in all sediment samples, despite the evident different tourist exploitation of the two examined beaches. Sediments of the most touristic beach had values between 729.5 ± 212.3 and 1327 ± 125.8 items/kg, instead, the less popular beach between 606.3 ± 102.8 and 1116.5 ± 226.9 items/kg (average and st. dev). Microparticle abundance varied before and after the touristic summer season, increasing in the most popular beach and decreasing in the unpopular one. Differences in microparticle abundance between foreshore and backshore were present too; however, statistical analyses did not show evident relations between microparticle abundance and the distance from the see. Grain size influenced the abundance of microparticles in sediments. Our results improve knowledge on microparticle pollution in marine environments, highlighting information about micropollution in coastal areas. Future studies are needed to understand better microparticle dynamics and ecological impacts in marine and terrestrial systems, implementing new strategies to monitor pollution state, enhancing the natural intermediate environments, and providing useful and sustainable measure of conservation.

**Supplementary Information:**

The online version contains supplementary material available at 10.1007/s10661-024-13159-z.

## Introduction

Marine ecosystems are constantly threated by anthropogenic activities and litter, especially along the coasts (Piazzolla et al., 2023). Any material of anthropogenic origin, abandoned or disposed of, in marine environment, from shores to deep oceans, can be consider marine litter, including all materials brought indirectly to the sea by rivers, sewage, and weather phenomena (Kershaw, 2016).

Plastic litter in marine environments is one of the most abundant and critical pollutant (Löhr et al., 2017; Sharma & Chatterjee, 2017). Benefits of plastics such as resistance, flexibility, durability, low cost, and low weight enabled the use of plastic materials in different products in the past decades. Unfortunately, when plastic waste reaches the environment, their characteristics make them extremely problematic pollutants in natural environments, especially for small-size particles. Plastic particles from 5 mm to 1 μm are considered microplastics (MPs) and can be produced with a small dimension (primary production) or as a result of the degradation of larger plastic objects (secondary production). MP pollution in marine ecosystems is problematic, especially because small fragmentation can increase food chain access and biota interactions (e.g., Assas et al., 2020; Devereux et al., 2021; Jahan et al., 2019; Marrone et al., 2021). Moreover, MPs pose a threat to coastal environments because they can adsorb chemicals and other pollutants (Frias et al., 2010; Lee et al., 2020; Li et al., 2018; Luo et al., 2019; Zhou et al., 2019). Different studies from all world regions report on MP occurrence in marine waters, sediments, and beaches (e.g., Bošković et al., 2021; Frias et al., 2010; Gholizadeh & Cera, 2022; La Daana et al., 2019). The zones between terrestrial and marine environments, such as beaches, are particularly vulnerable to litter pollution. Moreover, their recreational and touristic function enhance their exposure to pollution. Plastic is the most frequent type of litter in beaches (Balčiūnas & Blažauskas, 2014; Thiel et al., 2013). Fragmented plastics on beaches derived from inland sources, transported by water, wind, storms, human activity, or directly from the oceans, are transported also from great distances (Eriksen et al., 2014; Lebreton et al., 2012). Plastic materials on beaches are exposed to different environmental phenomena, such as abrasion due to wind, wave action, and sunlight, which facilitate plastic fragmentation into smaller pieces (Andrady, 2011). Water filtration due to hydraulic gradients resulting from wave action pushes particles from the surface into the sand body and is adsorbed onto the sand grain surfaces (Brown & McLachlan, 2010). Hence, sandy beaches act as a specific filtering system that captures organic and inorganic particles and MPs (Brown & McLachlan, 2010).

Unfortunately, the environment is not only contaminated by MPs. Pollution through anthropogenic fibers of cellulosic or animal origin is greatly underrepresented in the literature (Hasenmueller et al., 2023; Stanton et al., 2019; Suaria et al., 2020a, 2020b). However, recent research suggests that these particles can be as toxic for ecosystems as synthetic ones, due to chemical additives and dyes used during manufacturing processes (Athey & Erdle, 2022). In marine environments, MPs are considered the harmful fraction of wastes (Law & Thompson, 2014), being dangerous for organisms (Jahan et al., 2019; Ugwu et al., 2021), and potential carriers of pathogens and other pollutants (e.g., Li et al., 2018; Zhou et al., 2019); therefore, monitoring is fundamental to understand the relevance of the pollution. However, such as MPs, natural and regenerated fibers can pollute water and sediments and be ingested by biota; therefore, this kind of pollution cannot be neglected. Non-synthetic fibers are usually considered less dangerous in natural environments due to their biodegradability. However, little is known about their degradation in the marine environment, and some study highlighted a permanence of these materials for more than 130 years in the deep ocean (Athey & Erdle, 2022; Chen & Jakes, 2001). Moreover, their faster degradation in comparison to polymers could play a potential role in releasing toxic pollutants (Ladewig et al., 2015). Consequently, monitoring of microfibers (MFs) is of great interest to better understand microparticle pollution in marine environments.

The Mediterranean Sea is a semi-enclosed basin and, because of its environmental characteristics and hydrodynamics, become a wide area of accumulation of plastic debris and litter over time (Canals et al., 2021; Cózar et al., 2015; Eriksen et al., 2014; Lebreton et al., 2012; Pierdomenico et al., 2019). Previous works studied MP pollution in the Mediterranean area (e.g., Bošković et al., 2021; Digka et al., 2018), including Italy (e.g., Cannas et al., 2017; Guerranti et al., 2017). Marrone et al. (2021) analyzed the morphological characteristics and the polymeric composition of MPs collected from the sea surface in six stations of the Calabrian coasts, describing the different particle distribution from coastal areas up to 12 nautical miles offshore. This study showed evident differences in MP concentration between the Tyrrhenian (87%) and Ionian (13%) sides, due to the complex marine and atmospheric dynamics. However, only few research considered the presence of other micropollutants such as MFs (e.g., Suaria et al., 2020b).

Sandy beaches of Calabria are a popular touristic destination in Italy, especially during the summer period. In this study, two beaches with different tourist exploitation were sampled and analyzed, providing a more complete detection of micropollutants in the center of the Mediterranean Sea, evaluating the impact of tourism on microparticle accumulation and distribution, and if grain size affects the accumulation and distribution of microparticles within sediments (Alomar et al., 2016; Crawford & Quinn, 2016; Vermeiren et al., 2021). The aims of this study are (i) to quantify the abundance of microparticles of anthropogenic origin (MPs and MFs) in two nearby beaches, differing in their touristic exploitation (high versus low), (ii) to verify if microparticle abundance is higher after the main touristic summer season, and if it is more pronounced at the tourist beach than at the unpopular one, (iii) to verify if the accumulation of microparticles increases as the distance from the shore increases, and (iv) to verify if grain size influences the abundance of microparticles in sediments.

## Materials and methods

### Study area

**Fig. 1:**
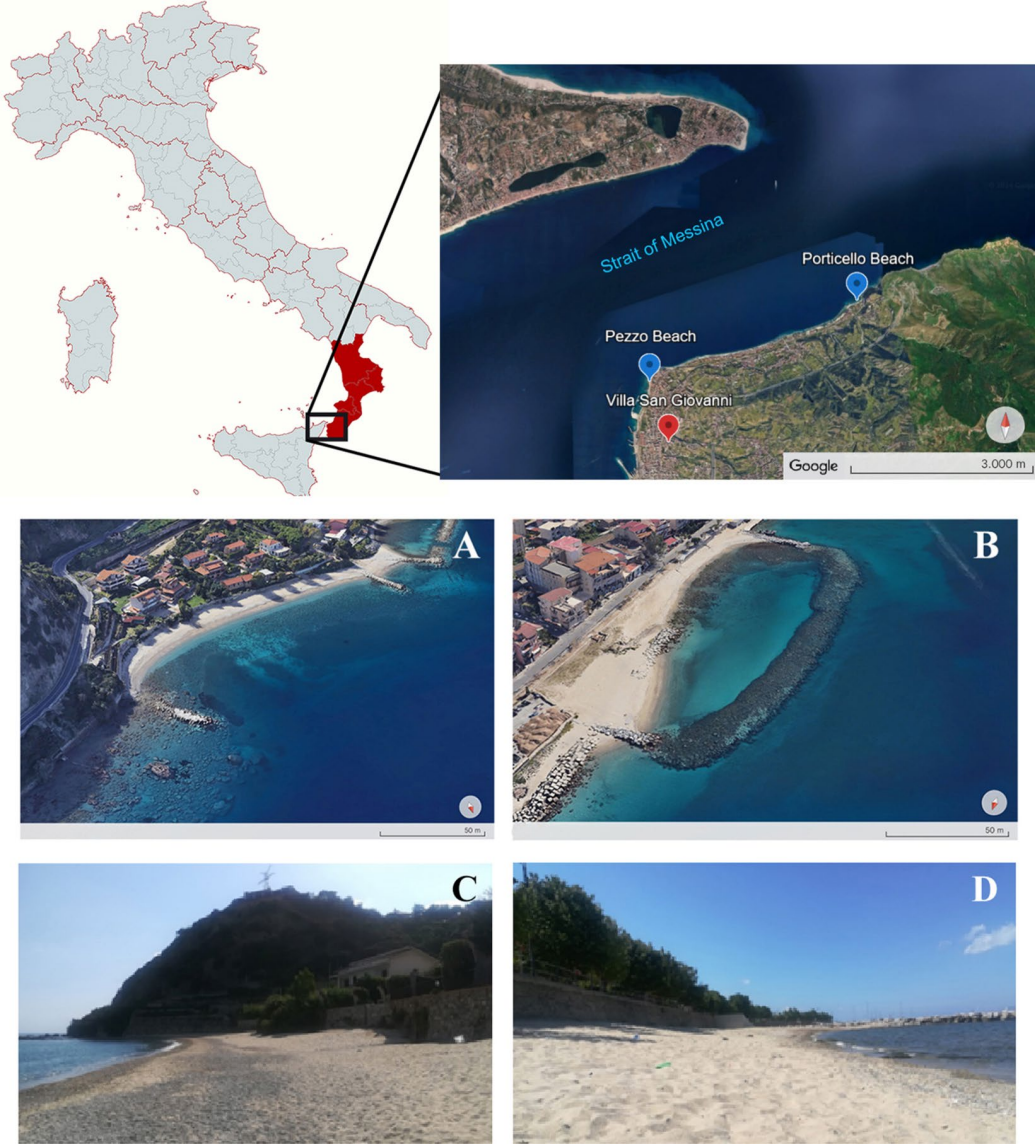
Location of the monitored beaches: Porticello **A**, **C** and Pezzo **B**, **D**. (Map of Italy created with mapchart.net; detail of Strait of Messina, images A and B from https://earth.google.com/, Imagery ©2023 Google, Airbus, Data SIO, NOAA, U.S. Navy, NGA, GEBCO, Map data ©2023, modified [access 2024–05-16]; photos C and D: F. Trunfio)

The Calabria region, Italy, is located in the center of the Mediterranean Sea, enclosed by the Tyrrhenian and the Ionian seas (Fig. 1). Calabria has a coastline of about 740 km. Regarding climatic characteristics, the Ionian and Tyrrhenian coasts are exposed to very different winds, causing a high variability of weather and sea conditions between the various coastal areas that influence coastal dynamics. The Strait of Messina separates peninsular Italy (Calabria) from the island of Sicily, connecting the Tyrrhenian and Ionian seas, two seas with different characteristics in terms of salinity and temperature (Barilla et al., 2021; Bignami & Salusti, 1990). The coasts of this stretch of sea are crossed by very strong currents, and the geomorphology of the beaches varies from year to year because of the strong winter storms, which could characterize a part of the pollution. The examined sites were chosen in this particular area, according to their location, their environmental characteristics, and human activities, in order to consider the influence of the anthropogenic impact.

The beaches of Porticello (38°14′33″N 15°40′27″E) and Pezzo (38°13′47″N 15°38′08″E) are located in the municipality of Villa San Giovanni, in the Reggio Calabria (RC) province. This coastal area is subject to pressure related to anthropogenic activities such as illegal fishing, widespread watercourse concreting, intense urbanization, maritime traffic, and road infrastructure (railways, viaducts, and tunnels) which has led to the discharge of large quantities of waste material into the sea (ARPACAL—Agenzia Regionale per la Protezione dell'Ambiente della Calabria 2016; MSFD - Marine Strategy Framework Directive, 2008). In addition, the local seabed is a landing point for submarine electricity cables and gas pipelines from Sicily. However, this Calabrian coastline is a protected area, with the aim of ensuring the long-term maintenance of natural habitats and flora and fauna species interesting at European level. The seabed is characterized by a steep bathymetric drop, reaching important depths (0–100 m) a few meters from the shore (ARPACAL—Agenzia Regionale per la Protezione dell'Ambiente della Calabria n.d.). Porticello and Pezzo beaches differ strongly in their geomorphology and in the frequencies of tourists they receive. Both sites are characterized by coarse sand near the sea and finer sand inwards. The beaches are mainly sandy but, in some cases, contain small proportions of gravel, usually on the lower part of the foreshore. At both beaches, beach cleaning is usually carried out by environmental associations once a year before the touristic season (April or May).

Porticello beach (Fig. 1A, C) overlooks the open sea segment to the north-west of the city of Villa San Giovanni and is characterized by a straight coastline. The beach is about 240 m long and 24 m wide, with a north west orientation. It is often subject to strong winds and sudden current change (ARPACAL—Agenzia Regionale per la Protezione dell'Ambiente della Calabria n.d.; Barilla et al., 2021). At Porticello beach, the prevailing wave motion arrival direction is transverse (inclination of about 45°); therefore, a groyne arranged orthogonally to the shoreline is present to protect the coastline, reducing coastal transport and intercept sediment. Although it is in a peripheric area compared to Pezzo beach, it is easily reached in 10 min by car from the city center. Therefore, this is an extremely crowded beach in the main tourist season (July and August). Moreover, the area is full of residences and houses very close to the sea, which are populated only in the summer months. At Porticello beach, the incident direction is transverse from the shoreline, which produces a movement of materials parallel to the coast and at the breakwater line.

Pezzo beach (Fig. 1B, D) is located inside the Strait of Messina and has a slightly concave configuration. It is 218 m long and has a variable width. At the sampling area, the beach is about 15 m wide. The wave regime characterizing this beach is more stable and less exposed to the wind than Porticello beach. The waves come perpendicularly to Pezzo beach, causing the removal of sediments and the erosion of the stretch of beach upstream. Therefore, a barrier construction parallel to the coast is present for its protection. The beach of Pezzo is 700 m from the port of Villa San Giovanni port; therefore, the sea area is affected by an intense traffic of merchant boats and passenger ships. This traffic favors the accumulation of marine litter along the coast, making this beach less frequented by tourists during the summer months.

### Sampling

**Fig. 2:**
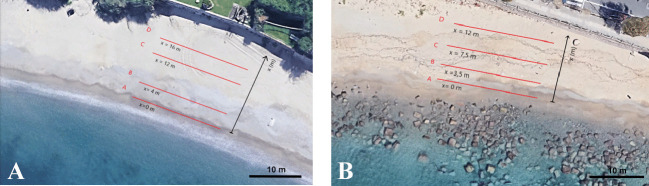
Sampling transects in Porticello **A** and Pezzo **B** beaches. (Images from https://earth.google.com/, Imagery ©2022 Google, Data SIO, NOAA, U.S. Navy, NGA, GEBCO, Landsat/Copernicus, Map data ©2022, modified)

Sampling methodology was performed taking as a reference the guidelines described by the European Commission (2013) for MP monitoring. Samplings were carried out once before and once after the summer period, in June and September 2021. A total of eight samples were collected at each beach (Porticello and Pezzo), four in June and four in September. Four sampling transects perpendicular to the coastline, two on the foreshore and two on the backshore, with a length of 20 m, were defined at each beach (Fig. 2). The width of each transect varied depending on the total width of each beach (Fig. 2), covering a total sampling area of 240 m^2^ for Porticello beach and 180 m^2^ for Pezzo beach. Four sampling spots were made for each transect, every 5 m. For each sampling point, two sediment collections of about 7–8 g were made: the first on the beach surface (about first 5 cm) and the second one at about 10–15 cm depth, after removing surface sediments. Finally, samples from the two foreshore transects (A and B in Fig. 2) and the two backshore transects (C and D in Fig. 2) were combined to highlight the differences between the shoreline and the backshore. Each sample was about 225 g.

Sediment samples were collected in glass jars using a metal spoon and stored in a fridge at 15 °C until laboratory analysis. During all steps nitrile gloves and cotton clothing were worn. The sampling sequence was carried out by moving along the reference transects in the opposite wind direction to avoid contamination.

### Laboratory analysis

To avoid contamination during laboratory processing, plastic materials were replaced with glass and metal equipment, when possible, and all working surfaces and all laboratory glassware were cleaned with ethanol and Milli-Q water during all steps. Nitrile gloves were used by researchers during all steps, together with cotton coats. In accordance with Cabrera et al. (2020), abrasive substances were not used, reducing the preparation time and decreasing the risk of pollutant contamination.

Samples were placed in an aluminum box, covered with aluminum foil and dried in an oven at 40 °C to constant weight. Sediments were sieved for granulometric classification with a mechanical sifter for 3 min, intensity of impulses 6/9. Sediments were sifting using sieves with 1 and 0.5 mm of mesh, following the typical sand separation classes described in Crawford and Quinn (2016): > 1 mm very coarse sand, 1–0.5 mm coarse sand, < 0.5 mm medium, and fine sand). At once, this separation was useful for separating big microparticles (5–1 mm) from the small fraction, and the small fraction in two subsamples. A 1:1 15% H₂O₂ solution was used for the organic matter removal (OMR) step. Finally, treated sediments were dried in the oven at 40 °C to constant weight. NaCl solution (200 g NaCl/0.6 L, density 1.2) was added to dried sediments and blended with a magnetic mixer for 2 min, as suggested in Balestra and Bellopede (2022). Density separation with NaCl for sediment samples could limit the ability to capture anthropogenic materials with higher densities; however, this solution is eco-friendly, and the density of materials left in natural environments is not necessary the same as newly one. Material porosity, degradation, and organic activity can increase or decrease their density in natural environments (Kaiser et al., 2017); therefore, not only the “lightest” fraction of anthropogenic litter could be extracted with this solution. Samples were left to rest for 24 h, favoring sediment deposition. The supernatant was extracted with a glass pipet and filtered through a 1.2-μm pore size glass microfiber filter (Whatman, Ø 47 mm). Filters were placed on glass petri dishes, covered with aluminum foil to avoid air contamination, and dried into an oven at 40 °C.

### Microparticle identification and characterization

MPs and MFs can be detected with visual identification under a microscope (e.g., Alomar et al., 2016; Guerranti et al., 2017; Houck, 2009; Khan et al., 2017; Mathalon & Hill, 2014; Zhang, 2014), being an inexpensive methodology (Crawford & Quinn, 2016). However, it does not allow to identify the chemical composition of materials. Fluorescent additives are often used in plastic and textile production worldwide, especially whitening agents (Qiu et al., 2015); therefore, many materials can be easily observed under ultraviolet (UV) light (Balestra & Bellopede, 2023; Balestra et al., 2023; Ehlers et al., 2020; Klein & Fischer, 2019).

In this work, microparticles on filters were observed with and without a Alonefire SV10 365 nm UV flashlight 5W under a Leitz ORTHOLUX II POL-MK microscope equipped with a DeltaPix Invenio 12EIII 12 Mpx Camera, starting with 2.5 × zoom, increased to 10 × or more for fiber identification, as described in Balestra and Bellopede (2022) (Supplementary Fig. 1). Visual identification was used to count MPs according to the strict selection criteria described in Crawford and Quinn (2016). Images of natural, regenerated, and synthetic fibers under a microscope taken in previous works were used for MF comparisons (e.g., Houck, 2009; Khan et al., 2017; Zhang, 2014). Particles smaller than 0.1 mm and those that were not clearly identifiable were not take into account (European Commission 2013).

The software PAST version 2.17c (Hammer et al., 2001) was used to perform statistical analyses. A chi-squared test was used to check if considered variables were related or independent.

## Results

**Fig. 3:**
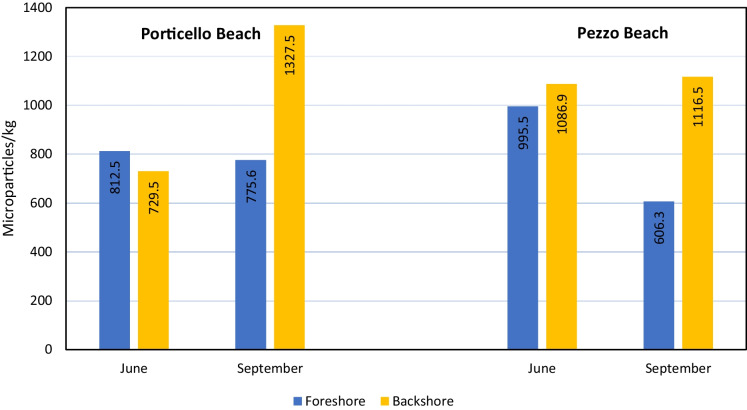
Weighted averages of microparticle concentrations of anthropogenic origin in relation to the distance from the see in Porticello and Pezzo beaches, before (June) and after (September) the tourist season

The grain size of the three main classes (> 1 mm, 1–0.5 mm, and < 0.5 mm) of the analyzed sediments are reported in Fig. 3 and Supplementary Table 1. Porticello Beach foreshore and backshore were characterized especially by coarse sand, while in Pezzo Beach was mainly present coarse sand in foreshore and medium-fine sand in backshore.

MPs and MFs were found in all examined samples, in both beaches (Fig. 3). The 58% of the examined microparticles were MPs, highlighting a significant percentage of natural and regenerated anthropogenic fibers polluting marine environment. Weighted averages of microparticle concentrations in relation to the distance from the sea, before and after the tourist season, are reported in Fig. 3 and Supplementary Table 2.

Statically significant differences in microparticle abundance between June and September were present in both beaches (*χ*^2^ = 114.96, *p* < 0.01). Microparticle abundance varied before and after the summer season, relevantly increasing in Porticello beach, the most popular, and decreasing in Pezzo beach.

Microparticle abundance considering both beaches was independent from the sea distance (foreshore/backshore) (*χ*^2^ = 1.65, *p* = 0.2). However, Porticello beach doubled microparticles abundance in the backshore from June to September, while in the foreshore, the microparticles abundance decreased slightly. The difference between the amount of microparticles found in foreshore and backshore, from June to September, is statistically significant (*χ*^2^ = 90.48, *p* < 0.01). In Pezzo beach, microparticles abundance decreased of about one-third from June to September, while in the backshore, it slightly rise. The difference between the amount of microparticles found in foreshore and backshore, from June to September, is statistically significant (*χ*^2^ = 61.53, *p* < 0.01).

**Fig. 4:**
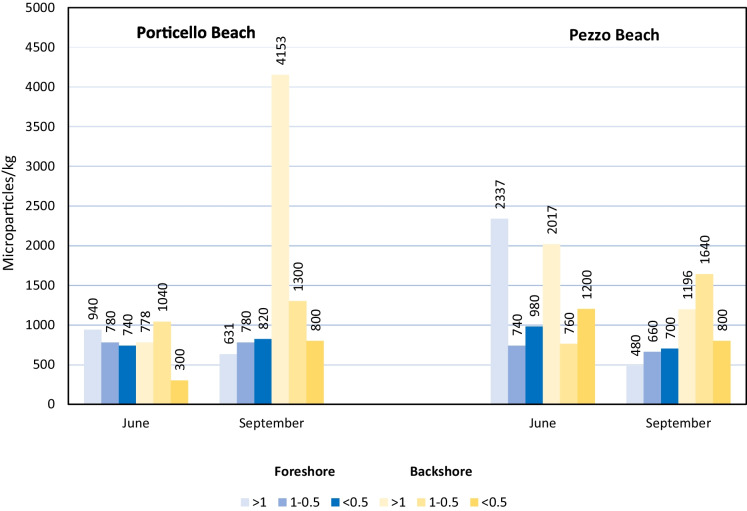
Abundance of microparticles of anthropogenic origin in Porticello and Pezzo beaches, before and after the tourist season. Data in relation to sediment grain size

Relation between the abundance of microparticles and the sediment grain size was found considering both beaches (*χ*^2^ = 175.67, *p* < 0.01), Porticello beach (*χ*^2^ = 1015.70, *p* < 0.01) and Pezzo beach (*χ*^2^ = 100.65, *p* < 0.01) (Fig. 4). There was also a statistical relation between microparticle abundance from June to September and the sediment grain size for both Porticello (*χ*^2^ = 462.93, *p* < 0.01) and Pezzo beaches (*χ*^2^ = 1036.20, *p* < 0.01) (Fig. 4).

## Discussion

### Considerations about methodology

Research on MPs and MFs is challenging. Different methods for MP determination exist; however, there is no general consensus on MP definition or methodologies. Moreover, MF methodology are often the same of MPs (Frias & Nash, 2019; International Organization for Standardization and European Committee for Standardization 2020).

Avoiding contamination during sampling and laboratory analysis is difficult; however, plastic materials were avoided when possible, and all working surfaces and laboratory glassware were cleaned with ethanol and Milli-Q water during all steps. Nitrile gloves were used by researchers during sampling and analysis. Not placing control filters in the laboratory could prevent the recognition of some other contamination that the samples could have suffered during all procedures; however, laboratory analysis were made under the hood.

The sediment subdivision into grain size classes made it possible to examine the samples in a more representative manner, allowing to do considerations and correlations between microparticles abundances and the size of the sediments.

Marine and beach sediment samples contain organic matter that may interfere with the analyses changing the apparent density of polymers and making easier aggregation with microparticles (Eerkes-Medrano et al., 2015; Morét-Ferguson et al., 2010). Therefore, OMR is a fundamental step for MP and MF analyses. The most commonly used chemicals are HCl, HNO₃, NaOH, KOH, and especially H₂O₂. A 30% H₂O₂ solution can damage or dissolve smaller MPs (Nuelle et al., 2014); however, 15% H₂O₂ cannot remove all organic materials. OMR on environmental samples should be done in relation to the sample characteristics; therefore, preliminary observations on samples must be done. The choice of treatment for OMR depends on the examined matrix, and several protocols are present in literature (e.g., Corami et al., 2020; Crawford & Quinn, 2016; Mukhanov et al., 2019); however, there is no standardized methodology. An incorrect OMR choice may result in the partial or complete degradation of non-synthetic MFs, resulting in counting errors, under- or overstimation (Athey & Erdle, 2022; Duis & Coors, 2016; Nuelle et al., 2014; Rocha-Santos & Duarte, 2015; Treilles et al., 2020). Currently, H₂O₂ is one of the most common methods used for OMR in MP and MF analyses (Athey & Erdle, 2022); however, it can affect particle properties and spectroscopic analyses, increasing the fragility of different kinds of materials. Microparticles with fluorescent whitening agents are easily detectable under UV light; nevertheless, a lot of organic and inorganic materials are fluorescent under UV light too; therefore, OMR is a fundamental step before this kind of detection.

For sediment samples, density separation with NaCl could limit the ability to capture anthropogenic materials with higher densities; however, this solution is eco-friendly and the density of materials left in natural environments is not necessary the same as newly one. The specific density of certain plastics is higher, and in different cellulosic materials, they have higher density than the one of several synthetic polymers, such as polyester, polypropylene, and nylon/acrylics. However, material porosity, degradation, and organic activity can increase or decrease their density in natural environments (Kaiser et al., 2017); therefore, not only the “lightest” fraction of anthropogenic litter could be extracted with this solution. However, it should be taken into account that high-density materials can remain into the sediments.

A combination of microscopy and spectroscopy methods is probably the optimal choice to characterize MPs and MFs in natural matrices. Microscopy is faster than spectroscopy; however, it does not allow to identify microparticle composition. Nevertheless, during spectroscopic analyses, spectra obtained are difficult to match with high percentages with those of the libraries because surface of microparticles remained in natural environments for a long time is often degraded and contaminated by other materials (Song et al., 2015). Moreover, different cellulose-based fibers, natural and regenerated, have very similar chemical composition; therefore, it is difficult to differentiate them with spectroscopic analysis. Microscopy can be more helpful in identify these materials (Peets et al., 2017). In both microscopic and spectroscopic analysis, the overestimation or underestimation of microparticles has been observed (e.g., Hidalgo-Ruz et al., 2012; Song et al., 2015), and both methods are susceptible to operator biases and errors. The purpose of these work was not to feature microparticles characteristics, but to estimate microparticle abundances and discover their movements related to environmental characteristics and tourism on the monitored beaches, microscopic analyses following the strict criteria described in Crawford and Quinn (2016) are enough.

### Considerations on microplastic and microfiber pollution

In this study, microparticles were found in all collected samples, highlighting an intense pollution. Microparticle abundance varied before and after the touristic summer season, relevantly increasing in Porticello beach, the most popular, and decreasing in Pezzo one, probably given its proximity to the Villa San Giovanni port. Significant differences in microparticle contamination between the two sites could depend on the direct anthropogenic influence, as the seaside tourism, or by indirect pollution. The Mediterranean Sea is characterized by a complex marine circulation which favor pollution in the semi-enclosed basin (Cózar et al., 2015; Eriksen et al., 2014; Lebreton et al., 2012), and the prevailing superficial winds blow contribute to the accumulation of floating pollutants toward the Tyrrhenian coasts. Therefore, it is reasonable to assume that the microparticle accumulation was also linked to the complex marine and atmospheric currents circulation. Comparisons with other researches are particularly difficult as there is no standardized method and a lot of studies consider only MPs. However, some similarities can be observed. In Vermeiren et al. (2021), two Uruguayan sites were analyzed, one with a high anthropogenic impact and one with a lower one, showing a high number of MPs (5 mm to 66 μm) in the most touristic area, as well as in our study. Sediment samples collected outside Talamone harbor, Italy, an area characterized by low coast, consisting of sandy loam and/or silty clay deposits highlighted MP abundances from 62 ± 24 since 466 ± 297 particles/kg. Whereas 58% of our data were MPs, the abundances found in our examined Calabrian beaches were only slightly higher respect to those found in the area of Talamone.

With regard to spatial distribution, there is a tendency for objects to settle closer to dunes or creeks, which degrading lead to the formation of micro litter. Our results show that microparticle accumulation is greater towards the back of the beach, with the exception of Porticello beach in June; however, statistical analyses did not show evident relations. The shore area in which the waves break is an accumulation zone for micro litter coming from the sea. Therefore, sediment sampling in this area could lead to an overestimation during micro litter analyses (Merlino et al., 2020). Moreover, the distribution of micro litters and in this area is highly variable depending on the climate variations. Instead, the micro litter accumulation in the inner part of the beach is static, due to the transport by the action of high tide and wind to the backshore. High abundances of MPs in this area were found in different work, including in the deep layers (Hidalgo-Ruz & Thiel, 2013; Turra et al., 2014). However, other research show discrepancies by assuming that MP distribution along the beaches can vary depending on the sampling area, the monitoring period, and the morphology of the examined site (Vermeiren et al., 2021). Each sample is representative of the time at which it was taken. Sampling before a substantial accumulation of debris along the shore, for example, after a swell, can affect the results, highlighting excessive pollution. In Porticello beach, in the month of June, the amount of microparticles found in the foreshore could be higher than the backshore due to the rough seas in the days before the sampling. The accumulation of microparticles at the high tide line depends on the force of the wave motion and the tidal cycle (Hinata et al., 2017; Lee et al., 2015; Moreira et al., 2016). At both sites, the wave breakwater line was about 2–3 m from the low tide line. The arrival of the wave direction on the beach is different for the two sampling sites, affecting microparticle accumulation. Moreover, in both beaches, artificial structures were present to avoid costal erosion. At Porticello beach, the incident wave direction is transverse from the shoreline, which, together with the groyne, favors accumulation of materials. At Pezzo beach, the wave motion comes in a direction perpendicular to the shore, favoring erosion; therefore, a barrier construction parallel to the coast is present for its protection. In Marrone et al. (2021), small plastic particles ranging from 5 mm to 50 μm were collected from the sea surface of different Calabrian coasts, highlighting a MP density of 0.13 ± 0.19 particles/m^2^. Other studies in marine waters of the Mediterranean area showed a density of 0.243 particles/m^2^ (Cózar et al., 2015), 0.40 ± 0.74 particles/m^2^ (Suaria et al., 2016).

Sediment samples collected along transepts of varying length and width, parallel or perpendicular to the coastline, do not allow the origin of the debris to be traced; therefore, it is not possible to know whether the source of the pollution is from terrestrial sources, if they are transported from the sea by floating on the surface or by transport on the bottom. Additionally, it is not possible to estimate a global distribution framework that satisfies well-defined criteria because the weather-sea climate and the variation of currents have a significant impact on the rate of debris accumulation (Bergmann et al., 2015). Glass and heavy plastics seem to accumulate mainly on rocky beaches (Moore et al., 2001), while the lighter micro-litter accumulate along coasts not affected by strong wind, which would otherwise favor their off-shore spread (Costa et al., 2011).

Relation between the abundance of microparticles and the sediment grain size was found for our examined beaches. Vermeiren et al. (2021) showed a significant correlation between the abundance of microparticles and the size of the sediments in which they were found too. In contrast, in Alomar et al. (2016), no correlation was found, highlighting also that a finer sediment fraction is not always associated with a greater amount of MPs.

## Conclusions

Sandy beaches of Italy are a popular touristic destination during the summer period and, therefore, may be more threatened by pollution. Most studies in natural environment focused on MPs only, neglecting an important component of anthropogenic microfiber pollution. In this study, one popular and heavily used beach of Calabria region against an unpopular one was monitored for conservation purposes, improving knowledge on microparticles of anthropogenic origin (microplastics and microfibers).

Microparticles were found with high amount in all examined sediment samples, despite the differences in touristic exploitation of the two beaches. Microparticle abundance varied before and after the summer season, increasing in the most touristic beach and decreasing in the unpopular one. Differences between foreshore and backshore microparticle abundances were present; however, statistical analyses did not show evident relations. A relation between grain size and micro litter abundance was highlighted. These results suggest that micropollutant accumulation processes are not depending on the effect of tourism and/or urbanization only, highlighting the important role of the complex marine and atmospheric circulation in structuring beach microparticle pollution.

Microparticle monitoring is the first step to understand the health status of the marine environment, possible threats, sources, and effects of micropollutant of anthropogenic origin. In marine environments, MPs are considered the harmful fraction of wastes; however, such as MPs, natural, and regenerated fibers can pollute water, sediments, and be ingested by biota; therefore, this kind of pollution cannot be neglected. Monitoring is fundamental to understand the relevance of the pollution and the environmental sustainability. Future extensive monitoring programs are needed to better understand dynamics, transport, ecological impacts, and degradation rates of microparticles of anthropogenic origin in the marine and terrestrial systems. Greater efforts should be made to protect marine ecosystems and their resources, implementing new strategies to monitor and mitigate pollution and providing useful solution and an adequate environmental education following the sustainability principles.

## Supplementary information

Below is the link to the electronic supplementary material.

Supplementary file1 (DOCX 626 KB)

## Data Availability

No datasets were generated or analysed during the current study.

## References

[CR1] Alomar, C., Estarellas, F., & Deudero, S. (2016). Microplastics in the Mediterranean Sea: Deposition in coastal shallow sediments, spatial variation and preferential grain size. *Marine Environment Research,**115*, 1–10. 10.1016/j.marenvres.2016.01.005 10.1016/j.marenvres.2016.01.005 26803229

[CR2] Andrady, A. L. (2011). Microplastics in the marine environment. *Marine Pollution Bulletin,**62*(8), 1596–1605. 21742351 10.1016/j.marpolbul.2011.05.030

[CR3] ARPACAL - Agenzia Regionale per la Protezione dell’Ambiente della Calabria (2016) Progetto dell’Unione Europea “Marine Strategy” - Documentazione sottoregione “Mediterraneo Centrale - Ionio”. https://www.arpacal.it/index.php/24-tematiche-ambientali/balneazione?start=256. Accessed 09 September 2024

[CR4] ARPACAL - Agenzia Regionale per la Protezione dell’Ambiente della Calabria (n.d.) Fondali da Punta Pezzo a Capo dell’Armi. https://sic.arpacal.it/fondali-punta-pezzo/. Accessed 09 September 2024

[CR5] Assas, M., Qiu, X., Chen, K., Ogawa, H., Xu, H., Shimasaki, Y., & Oshima, Y. (2020). Bioaccumulation and reproductive effects of fluorescent microplastics in medaka fish. *Marine Pollution Bulletin,**158*, 111446. 10.1016/j.marpolbul.2020.111446 32753222 10.1016/j.marpolbul.2020.111446

[CR6] Athey, S. N., & Erdle, L. M. (2022). Are we underestimating anthropogenic microfiber pollution? A critical review of occurrence, methods, and reporting. *Environmental Toxicology and Chemistry,**41*(4), 822–837. 10.1002/etc.5173 34289522 10.1002/etc.5173

[CR7] Balčiūnas, A., & Blažauskas, N. (2014). Scale, origin and spatial distribution of marine litter pollution in the Lithuanian coastal zone of the Baltic Sea. *Baltica,**27*, 39. 10.5200/baltica.2014.27.14

[CR8] Balestra, V., & Bellopede, R. (2022). Microplastic pollution in show cave sediments: First evidence and detection technique. *Environmental Pollution,**292*, 118261. 10.1016/j.envpol.2021.118261 34601031 10.1016/j.envpol.2021.118261

[CR9] Balestra, V., & Bellopede, R. (2023). Microplastics in caves: A new threat in the most famous geo-heritage in the world. Analysis and comparison of Italian show caves deposits. *Journal of Environmental Management,**342*, 118189. 10.1016/j.jenvman.2023.118189 37210820 10.1016/j.jenvman.2023.118189

[CR10] Balestra, V., Vigna, B., De Costanzo, S., & Bellopede, R. (2023). Preliminary investigations of microplastic pollution in karst systems, from surface watercourses to cave waters. *Journal of Contaminant Hydrology,**252*, 104117. 10.1016/j.jconhyd.2022.104117 36424222 10.1016/j.jconhyd.2022.104117

[CR11] Barilla GC, Barbaro G, Foti G, Mancuso P, Fiamma V, Malesinska A, Puntorieri P, Mandalari M Coastal erosion hazard and vulnerability: Case study of Porticello, South Calabria, Italy. In: Proceedings of 11th International Conference on Sustainable Water Resources Management: Effective Approaches for River Basins and Urban Catchments, WRM, 2021. 181–193

[CR12] Bergmann M, Gutow L, Klages M (2015) Marine anthropogenic litter. Springer Nature

[CR13] Bignami F, Salusti E (1990) Tidal currents and transient phenomena in the Strait of Messina: A review. . In: Pratt LJ (ed) The physical oceanography of sea straits, vol 318. NATO ASI Series. Springer, Dordrecht. 10.1007/978-94-009-0677-8_4

[CR14] Bošković, N., Joksimović, D., Peković, M., Perošević-Bajčeta, A., & Bajt, O. (2021). Microplastics in surface sediments along the Montenegrin Coast, Adriatic Sea: Types, occurrence, and distribution. *Journal of Marine Science and Engineering,**9*(8), 841. 10.3390/jmse9080841

[CR15] Brown, A. C., & McLachlan, A. (2010). *The ecology of sandy shores*. Elsevier.

[CR16] Cabrera, M., Valencia, B. G., Lucas-Solis, O., Calero, J. L., Maisincho, L., Conicelli, B., Moulatlet, G. M., & Capparelli, M. V. (2020). A new method for microplastic sampling and isolation in mountain glaciers: A case study of one antisana glacier, Ecuadorian Andes. *Case Stud Chem Environ Eng,**2*, 100051. 10.1016/j.cscee.2020.100051

[CR17] Canals, M., Pham, C. K., Bergmann, M., Gutow, L., Hanke, G., Van Sebille, E., Angiolillo, M., Buhl-Mortensen, L., Cau, A., & Ioakeimidis, C. (2021). The quest for seafloor macrolitter: A critical review of background knowledge, current methods and future prospects. *Environmental Research Letters,**16*(2), 023001. 10.1088/1748-9326/abc6d4

[CR18] Cannas, S., Fastelli, P., Guerranti, C., & Renzi, M. (2017). Plastic litter in sediments from the coasts of south Tuscany (Tyrrhenian Sea). *Marine Pollution Bulletin,**119*(1), 372–375. 10.1016/j.marpolbul.2017.04.008 28410785 10.1016/j.marpolbul.2017.04.008

[CR19] Chen, R., & Jakes, K. A. (2001). Cellulolytic biodegradation of cotton fibers from a deep-ocean environment. *Journal of the American Institute for Conservation,**40*(2), 91–103. 10.1179/019713601806113076

[CR20] Corami, F., Rosso, B., Bravo, B., Gambaro, A., & Barbante, C. (2020). A novel method for purification, quantitative analysis and characterization of microplastic fibers using Micro-FTIR. *Chemosphere,**238*, 124564. 10.1016/j.chemosphere.2019.124564 31472348 10.1016/j.chemosphere.2019.124564

[CR21] Costa M, Silva-Cavalcanti J, Barbosa C, Portugal J, Barletta M (2011) Plastics buried in the inter-tidal plain of a tropical estuarine ecosystem. Journal of Coastal Research 339–343

[CR22] Cózar, A., Sanz-Martín, M., Martí, E., González-Gordillo, J. I., Ubeda, B., Gálvez, J. Á., Irigoien, X., & Duarte, C. M. (2015). Plastic accumulation in the Mediterranean Sea. *PLoS ONE,**10*(4), e0121762. 10.1371/journal.pone.0121762 25831129 10.1371/journal.pone.0121762 PMC4382178

[CR23] Crawford CB, Quinn B (2016) Microplastic pollutants. Elsevier Amsterdam

[CR24] Devereux, R., Hartl, M. G., Bell, M., & Capper, A. (2021). The abundance of microplastics in cnidaria and ctenophora in the North Sea. *Marine Pollution Bulletin,**173*, 112992. 10.1016/j.marpolbul.2021.112992 34649204 10.1016/j.marpolbul.2021.112992

[CR25] Digka N, Tsangaris C, Kaberi H, Adamopoulou A, Zeri C Microplastic abundance and polymer types in a Mediterranean environment. In: Proceedings of the international conference on microplastic pollution in the Mediterranean Sea, 2018. Springer, 17–24. 10.1007/978-3-319-71279-6_3

[CR26] Duis, K., & Coors, A. (2016). Microplastics in the aquatic and terrestrial environment: Sources (with a specific focus on personal care products), fate and effects. *Environmental Sciences Europe,**28*(1), 1–25. 10.1186/s12302-015-0069-y 27752437 10.1186/s12302-015-0069-y PMC5044952

[CR27] Eerkes-Medrano, D., Thompson, R. C., & Aldridge, D. C. (2015). Microplastics in freshwater systems: A review of the emerging threats, identification of knowledge gaps and prioritisation of research needs. *Water Research,**75*, 63–82. 10.1016/j.watres.2015.02.012 25746963 10.1016/j.watres.2015.02.012

[CR28] Ehlers, S. M., Maxein, J., & Koop, J. H. (2020). Low-cost microplastic visualization in feeding experiments using an ultraviolet light-emitting flashlight. *Ecological Research,**35*(1), 265–273. 10.1111/1440-1703.12080

[CR29] Eriksen, M., Lebreton, L. C., Carson, H. S., Thiel, M., Moore, C. J., Borerro, J. C., Galgani, F., Ryan, P. G., & Reisser, J. (2014). Plastic pollution in the world’s oceans: More than 5 trillion plastic pieces weighing over 250,000 tons afloat at sea. *PLoS ONE,**9*(12), e111913. 10.1371/journal.pone.0111913 25494041 10.1371/journal.pone.0111913 PMC4262196

[CR30] European Commission (2013) Guidance on monitoring of marine litter in European seas. A guidance document within the common implementation strategy for the Marine Strategy Framework Directive. Ispra: European Commission, Joint Research Centre, MSFD Technical Subgroup on Marine Litter. 10.2788/99475

[CR31] Frias, J., Sobral, P., & Ferreira, A. M. (2010). Organic pollutants in microplastics from two beaches of the Portuguese coast. *Marine Pollution Bulletin,**60*(11), 1988–1992. 10.1016/j.marpolbul.2010.07.030 20800853 10.1016/j.marpolbul.2010.07.030

[CR32] Frias, J. P., & Nash, R. (2019). Microplastics: Finding a consensus on the definition. *Marine Pollution Bulletin,**138*, 145–147. 10.1016/j.marpolbul.2018.11.022 30660255 10.1016/j.marpolbul.2018.11.022

[CR33] Gholizadeh, M., & Cera, A. (2022). Microplastic contamination in the sediments of Qarasu estuary in Gorgan Bay, south-east of Caspian Sea Iran. *Science of The Total Environment,**838*, 155913. 10.1016/j.scitotenv.2022.155913 35569662 10.1016/j.scitotenv.2022.155913

[CR34] Guerranti, C., Cannas, S., Scopetani, C., Fastelli, P., Cincinelli, A., & Renzi, M. (2017). Plastic litter in aquatic environments of Maremma Regional Park (Tyrrhenian Sea, Italy): Contribution by the Ombrone river and levels in marine sediments. *Marine Pollution Bulletin,**117*(1–2), 366–370. 10.1016/j.marpolbul.2017.02.021 28202278 10.1016/j.marpolbul.2017.02.021

[CR35] Hammer Ø, Harper DA, Ryan PD (2001) PAST: Paleontological statistics software package for education and data analysis. Palaeontol Electron 4(1):9 http://palaeo-electronica.org/2001_1/past/issue1_01.htm

[CR36] Hasenmueller, E. A., Baraza, T., Hernandez, N. F., & Finegan, C. R. (2023). Cave sediment sequesters anthropogenic microparticles (including microplastics and modified cellulose) in subsurface environments. *Science of The Total Environment,**838*, 164690. 10.1016/j.scitotenv.2023.164690 10.1016/j.scitotenv.2023.164690 37302590

[CR37] Hidalgo-Ruz, V., Gutow, L., Thompson, R. C., & Thiel, M. (2012). Microplastics in the marine environment: A review of the methods used for identification and quantification. *Environmental Science and Technology,**46*(6), 3060–3075. 10.1021/es2031505 22321064 10.1021/es2031505

[CR38] Hidalgo-Ruz, V., & Thiel, M. (2013). Distribution and abundance of small plastic debris on beaches in the SE Pacific (Chile): A study supported by a citizen science project. *Marine Environment Research,**87*, 12–18. 10.1016/j.marenvres.2013.02.015 10.1016/j.marenvres.2013.02.015 23541391

[CR39] Hinata, H., Mori, K., Ohno, K., Miyao, Y., & Kataoka, T. (2017). An estimation of the average residence times and onshore-offshore diffusivities of beached microplastics based on the population decay of tagged meso-and macrolitter. *Marine Pollution Bulletin,**122*(1–2), 17–26. 10.1016/j.marpolbul.2017.05.012 28624357 10.1016/j.marpolbul.2017.05.012

[CR40] Houck MM (2009) Identification of textile fibers vol 84. Woodhead Publishing in Textiles

[CR41] International Organization for Standardization, European Committee for Standardization (2020) Plastics - Environmental aspects - State of knowledge and methodologies (CEN ISO/TR 21960:2020).

[CR42] Jahan, S., Strezov, V., Weldekidan, H., Kumar, R., Kan, T., Sarkodie, S. A., He, J., Dastjerdi, B., & Wilson, S. P. (2019). Interrelationship of microplastic pollution in sediments and oysters in a seaport environment of the eastern coast of Australia. *Science of the Total Environment,**695*, 133924. 10.1016/j.scitotenv.2019.133924 31756867 10.1016/j.scitotenv.2019.133924

[CR43] Kaiser, D., Kowalski, N., & Waniek, J. J. (2017). Effects of biofouling on the sinking behavior of microplastics. *Environmental Research Letters,**12*(12), 124003. 10.1088/1748-9326/aa8e8b

[CR44] Kershaw PJ (2016) Marine plastic debris and microplastics–Global lessons and research to inspire action and guide policy change

[CR45] Khan, A., Abir, N., Rakib, M. A. N., Bhuiyan, E. S., & Howlader, M. R. (2017). A review paper on textile fiber identification. *IOSR Journal of Polymer and Textile Engineering (IOSR-JPTE),**4*, 14–20.

[CR46] Klein, M., & Fischer, E. K. (2019). Microplastic abundance in atmospheric deposition within the Metropolitan area of Hamburg, Germany. *Science of the Total Environment,**685*, 96–103. 10.1016/j.scitotenv.2019.05.405 31174127 10.1016/j.scitotenv.2019.05.405

[CR47] La Daana, K. K., Johansson, C., Frias, J., Gardfeldt, K., Thompson, R. C., & O’Connor, I. (2019). Deep sea sediments of the Arctic Central Basin: A potential sink for microplastics. *Deep Sea Research Part i: Oceanographic Research Papers,**145*, 137–142. 10.1016/j.dsr.2019.03.003

[CR48] Ladewig, S. M., Bao, S., & Chow, A. T. (2015). Natural fibers: A missing link to chemical pollution dispersion in aquatic environments. *ACS Publications*. 10.1021/acs.est.5b04754 10.1021/acs.est.5b04754 26496674

[CR49] Law, K. L., & Thompson, R. C. (2014). Microplastics in the seas. *Science,**345*(6193), 144–145. 10.1126/science.1254065 25013051 10.1126/science.1254065

[CR50] Lebreton, L.-M., Greer, S., & Borrero, J. C. (2012). Numerical modelling of floating debris in the world’s oceans. *Marine Pollution Bulletin,**64*(3), 653–661. 10.1016/j.marpolbul.2011.10.027 22264500 10.1016/j.marpolbul.2011.10.027

[CR51] Lee, J., Lee, J. S., Jang, Y. C., Hong, S. Y., Shim, W. J., Song, Y. K., Hong, S. H., Jang, M., Han, G. M., & Kang, D. (2015). Distribution and size relationships of plastic marine debris on beaches in South Korea. *Archives of Environmental Contamination and Toxicology,**69*, 288–298. 10.1007/s00244-015-0208-x 26285904 10.1007/s00244-015-0208-x

[CR52] Lee, Y. K., Murphy, K. R., & Hur, J. (2020). Fluorescence signatures of dissolved organic matter leached from microplastics: Polymers and additives. *Environmental Science and Technology,**54*(19), 11905–11914. 10.1021/acs.est.0c00942 32852946 10.1021/acs.est.0c00942

[CR53] Li, J., Zhang, K., & Zhang, H. (2018). Adsorption of antibiotics on microplastics. *Environmental Pollution,**237*, 460–467. 10.1016/j.envpol.2018.02.050 29510365 10.1016/j.envpol.2018.02.050

[CR54] Löhr, A., Savelli, H., Beunen, R., Kalz, M., Ragas, A., & Van Belleghem, F. (2017). Solutions for global marine litter pollution. *Current Opinion in Environmental Sustainability,**28*, 90–99. 10.1016/j.cosust.2017.08.009

[CR55] Luo, H., Xiang, Y., He, D., Li, Y., Zhao, Y., Wang, S., & Pan, X. (2019). Leaching behavior of fluorescent additives from microplastics and the toxicity of leachate to Chlorella vulgaris. *Science of the Total Environment,**678*, 1–9. 10.1016/j.scitotenv.2019.04.401 31075575 10.1016/j.scitotenv.2019.04.401

[CR56] Marrone, A., La Russa, M. F., Randazzo, L., La Russa, D., Cellini, E., & Pellegrino, D. (2021). Microplastics in the center of mediterranean: Comparison of the two calabrian coasts and distribution from coastal areas to the open sea. *International Journal of Environmental Research and Public Health,**18*(20), 10712. 10.3390/ijerph182010712 34682461 10.3390/ijerph182010712 PMC8535489

[CR57] Mathalon, A., & Hill, P. (2014). Microplastic fibers in the intertidal ecosystem surrounding Halifax Harbor, Nova Scotia. *Marine Pollution Bulletin,**81*(1), 69–79. 10.1016/j.marpolbul.2014.02.018 24650540 10.1016/j.marpolbul.2014.02.018

[CR58] Merlino, S., Paterni, M., Berton, A., & Massetti, L. (2020). Unmanned aerial vehicles for debris survey in coastal areas: Long-term monitoring programme to study spatial and temporal accumulation of the dynamics of beached marine litter. *Remote Sensing,**12*(8), 1260. 10.3390/rs12081260

[CR59] Moore, S. L., Gregorio, D., Carreon, M., Weisberg, S. B., & Leecaster, M. K. (2001). Composition and distribution of beach debris in Orange County. *Marine Pollution Bulletin,**42*(3), 241–245. 10.1016/S0025-326X(00)00148-X 11381879 10.1016/s0025-326x(00)00148-x

[CR60] Moreira, F. T., Prantoni, A. L., Martini, B., de Abreu, M. A., Stoiev, S. B., & Turra, A. (2016). Small-scale temporal and spatial variability in the abundance of plastic pellets on sandy beaches: Methodological considerations for estimating the input of microplastics. *Marine Pollution Bulletin,**102*(1), 114–121. 10.1016/j.marpolbul.2015.11.051 26677755 10.1016/j.marpolbul.2015.11.051

[CR61] Morét-Ferguson, S., Law, K. L., Proskurowski, G., Murphy, E. K., Peacock, E. E., & Reddy, C. M. (2010). The size, mass, and composition of plastic debris in the western North Atlantic Ocean. *Marine Pollution Bulletin,**60*(10), 1873–1878. 10.1016/j.marpolbul.2010.07.020 20709339 10.1016/j.marpolbul.2010.07.020

[CR62] MSFD - Marine Strategy Framework Directive (2008) Marine Strategy (Direttiva Quadro 2008/56/CE; D.lgs. n.190/2010). https://strategiamarina.isprambiente.it/. Accessed 09 September 2024

[CR63] Mukhanov, V. S., Litvinyuk, D. A., Sakhon, E. G., Bagaev, A. V., Veerasingam, S., & Venkatachalapathy, R. (2019). A new method for analyzing microplastic particle size distribution in marine environmental samples. *Ecologica Montenegrina,**23*, 77–86. 10.37828/em.2019.23.10

[CR64] Nuelle, M.-T., Dekiff, J. H., Remy, D., & Fries, E. (2014). A new analytical approach for monitoring microplastics in marine sediments. *Environmental Pollution,**184*, 161–169. 10.1016/j.envpol.2013.07.027 24051349 10.1016/j.envpol.2013.07.027

[CR65] Peets, P., Leito, I., Pelt, J., & Vahur, S. (2017). Identification and classification of textile fibres using ATR-FT-IR spectroscopy with chemometric methods. *Spectrochimica Acta Part a: Molecular and Biomolecular Spectroscopy,**173*, 175–181. 10.1016/j.saa.2016.09.007 27643467 10.1016/j.saa.2016.09.007

[CR66] Piazzolla, D., Bonamano, S., De Muto, F., Scanu, S., Bernardini, S., Sodo, A., Della Ventura, G., & Marcelli, M. (2023). Microlitter occurrence, distribution, and summertime transport trajectories in the coastal waters of the north-eastern Tyrrhenian Sea (Italy). *Geosystems and Geoenvironment,**2*(4), 100192. 10.1016/j.geogeo.2023.100192

[CR67] Pierdomenico, M., Casalbore, D., & Chiocci, F. L. (2019). Massive benthic litter funnelled to deep sea by flash-flood generated hyperpycnal flows. *Science and Reports,**9*(1), 5330. 10.1038/s41598-019-41816-8 10.1038/s41598-019-41816-8 PMC6441077 30926913

[CR68] Qiu, Q., Peng, J., Yu, X., Chen, F., Wang, J., & Dong, F. (2015). Occurrence of microplastics in the coastal marine environment: First observation on sediment of China. *Marine Pollution Bulletin,**98*(1–2), 274–280. 10.1016/j.marpolbul.2015.07.028 26190791 10.1016/j.marpolbul.2015.07.028

[CR69] Rocha-Santos, T., & Duarte, A. C. (2015). A critical overview of the analytical approaches to the occurrence, the fate and the behavior of microplastics in the environment. *ChemistryTrAC Trends Anal Chem,**65*, 47–53. 10.1016/j.trac.2014.10.011

[CR70] Sharma, S., & Chatterjee, S. (2017). Microplastic pollution, a threat to marine ecosystem and human health: A short review. *Environmental Science and Pollution Research,**24*(27), 21530–21547. 10.1007/s11356-017-9910-8 28815367 10.1007/s11356-017-9910-8

[CR71] Song, Y. K., Hong, S. H., Jang, M., Han, G. M., Rani, M., Lee, J., & Shim, W. J. (2015). A comparison of microscopic and spectroscopic identification methods for analysis of microplastics in environmental samples. *Marine Pollution Bulletin,**93*(1–2), 202–209. 10.1016/j.marpolbul.2015.01.015 25682567 10.1016/j.marpolbul.2015.01.015

[CR72] Stanton, T., Johnson, M., Nathanail, P., MacNaughtan, W., & Gomes, R. L. (2019). Freshwater and airborne textile fibre populations are dominated by ‘natural’, not microplastic, fibres. *Science of the Total Environment,**666*, 377–389. 10.1016/j.scitotenv.2019.02.278 30798244 10.1016/j.scitotenv.2019.02.278

[CR73] Suaria, G., Achtypi, A., Perold, V., Lee, J. R., Pierucci, A., Bornman, T. G., Aliani, S., & Ryan, P. G. (2020). Microfibers in oceanic surface waters: A global characterization. *Science Advances,**6*(23), eaay8493. 10.1126/sciadv.aay8493 32548254 10.1126/sciadv.aay8493 PMC7274779

[CR74] Suaria, G., Avio, C. G., Mineo, A., Lattin, G. L., Magaldi, M. G., Belmonte, G., Moore, C. J., Regoli, F., & Aliani, S. (2016). The Mediterranean Plastic Soup: Synthetic polymers in Mediterranean surface waters. *Science and Reports,**6*(1), 37551. 10.1038/srep37551 10.1038/srep37551 PMC5120331 27876837

[CR75] Suaria G, Musso M, Achtypi A, Bassotto D, Aliani S Textile fibres in mediterranean surface waters: abundance and composition. In: Proceedings of the 2nd international conference on microplastic pollution in the mediterranean sea, 2020b. Springer, 62–66. 10.1007/978-3-030-45909-3_12

[CR76] Thiel, M., Hinojosa, I., Miranda, L., Pantoja, J., Rivadeneira, M., & Vásquez, N. (2013). Anthropogenic marine debris in the coastal environment: A multi-year comparison between coastal waters and local shores. *Marine Pollution Bulletin,**71*(1–2), 307–316. 10.1016/j.marpolbul.2013.01.005 23507233 10.1016/j.marpolbul.2013.01.005

[CR77] Treilles, R., Cayla, A., Gasperi, J., Strich, B., Ausset, P., & Tassin, B. (2020). Impacts of organic matter digestion protocols on synthetic, artificial and natural raw fibers. *Science of the Total Environment,**748*, 141230. 10.1016/j.scitotenv.2020.141230 32818900 10.1016/j.scitotenv.2020.141230

[CR78] Turra, A., Manzano, A. B., Dias, R. J. S., Mahiques, M. M., Barbosa, L., Balthazar-Silva, D., & Moreira, F. T. (2014). Three-dimensional distribution of plastic pellets in sandy beaches: Shifting paradigms. *Science and Reports,**4*(1), 4435. 10.1038/srep04435 10.1038/srep04435 PMC3967197 24670631

[CR79] Ugwu, K., Herrera, A., & Gómez, M. (2021). Microplastics in marine biota: A review. *Marine Pollution Bulletin,**169*, 112540. 10.1016/j.marpolbul.2021.112540 34087664 10.1016/j.marpolbul.2021.112540

[CR80] Vermeiren, P., Lercari, D., Munoz, C. C., Ikejima, K., Celentano, E., Jorge-Romero, G., & Defeo, O. (2021). Sediment grain size determines microplastic exposure landscapes for sandy beach macroinfauna. *Environmental Pollution,**286*, 117308. 10.1016/j.envpol.2021.117308 33991734 10.1016/j.envpol.2021.117308

[CR81] Zhang X (2014) Fundamentals of fiber science. DEStech Publications, Inc

[CR82] Zhou, Y., Liu, X., & Wang, J. (2019). Characterization of microplastics and the association of heavy metals with microplastics in suburban soil of central China. *Science of the Total Environment,**694*, 133798. 10.1016/j.scitotenv.2019.133798 31756811 10.1016/j.scitotenv.2019.133798

