# Supplementary material for: Microparticles of anthropogenic origin (microplastics and microfibers) in sandy sediments: A case study from calabria, italy
Source: Environ Monit Assess. 2024 Oct 1;196(10):993. doi: 10.1007/s10661-024-13159-z (PMC11445366; doi:10.1007/s10661-024-13159-z)
Supplement: Supplementary file 1 — Supplementary file1 (DOCX 626 KB) [file 10661_2024_13159_MOESM1_ESM.docx]

**SUPPLEMENTARY MATERIALS**

Microparticles of anthropogenic origin (microplastics and microfibres) in sandy sediments: a case study from Calabria, Italy

Valentina Balestra ^1,^*, Federica Trunfio ^2^, Sinem Hazal Akyıldız ^1^, Paola Marini ^1^ and Rossana Bellopede ^1^

^1^ Department of Environment, Land and Infrastructure Engineering (DIATI), Politecnico di Torino, Corso Duca degli Abruzzi 24, 10129 Torino, Italy

^2^ Politecnico di Torino, Corso Duca degli Abruzzi 24, 10129 Torino, Italy

* Correspondence: [valentina.balestra@polito.it](mailto:valentina.balestra@polito.it)

**SUPPLEMENTARY TABLES**

**Supplementary Table 1.** Grain size classes composition of the collected beach sediment samples. Percentages of the examined Porticello and Pezzo beaches sediment samples in June and September, divided in three main grain classes: >1 mm, 1-0.5 mm, and <0.5 mm.

|  | **Foreshore** |  | | **Backshore** |  | |
| --- | --- | --- | --- | --- | --- | --- |
| **Porticello beach** | **>1 mm** | **1-0.5 mm** | **<0.5 mm** | **>1 mm** | **1-0.5 mm** | **<0.5 mm** |
| June | 28.4 % | 39.5 % | 32.1% | 9.1 % | 52.1 % | 38.7 % |
| September | 13.2 % | 48.8 % | 38.1 % | 8.6 % | 47.7 % | 43.7 % |
| **Pezzo beach** | **>1** | **1-0.5** | **<0.5** | **>1** | **1-0.5** | **<0.5** |
| June | 10.2 % | 51.2 % | 38.6 % | 4.8 % | 34.7 % | 60.5 % |
| September | 33.1 % | 52.1 % | 14.8 % | 4.8 % | 35.4 % | 59.8 % |

**Supplementary Table 2.** Weighted averages of microparticle concentrations of anthropogenic origin in relation to the distance from the see in Porticello and Pezzo beaches, before (June) and after (September) the tourist season.

| **Porticello beach** | **Foreshore [items/Kg]** | **St.Dev.** | **Backshore [items/Kg]** | **St.Dev.** |
| --- | --- | --- | --- | --- |
| June | 812.5 | 29.1 | 729.5 | 212.3 |
| September | 775.6 | 127.2 | 1327.5 | 125.8 |
| **Pezzo beach** | **Foreshore [items/Kg]** | **St.Dev.** | **Backshore [items/Kg]** | **St.Dev.** |
| June | 995.5 | 66.2 | 1086.9 | 265.8 |
| September | 606.3 | 102.8 | 1116.5 | 226.9 |

**SUPPLEMENTARY FIGURES**

**
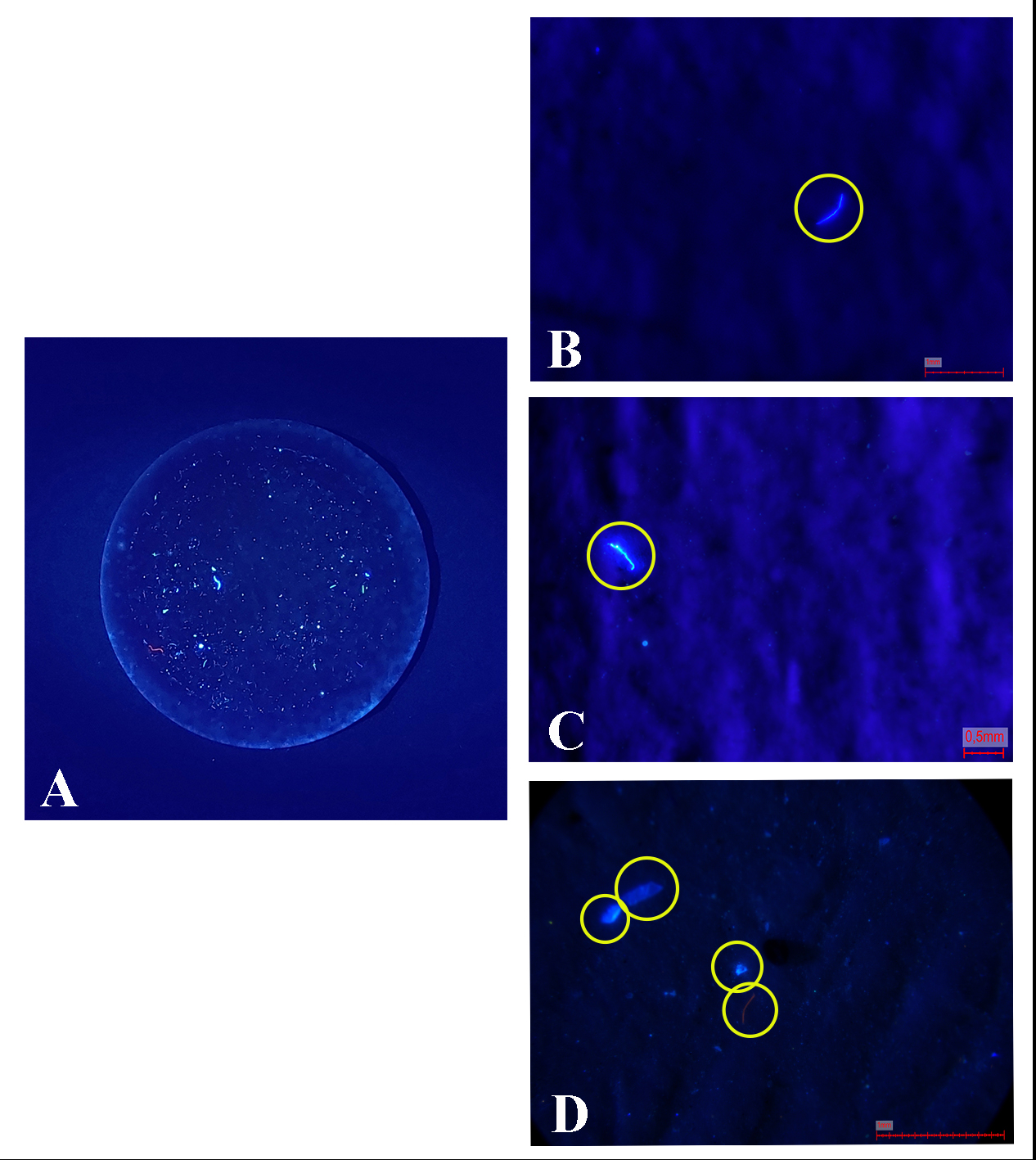
**

**Supplementary Figure 1.** Filter observed with UV light under microscope. A: Total filter; B, C, D: Details of microparticles with blue and red fluorescence.

**Porticello Beach**

**Supplementary Figure 2.** Grain size classes composition of the collected sediment samples of Porticello and Pezzo beaches.
